# Effects of metal-on-metal wear on the host immune system and infection in hip arthroplasty

**DOI:** 10.3109/17453674.2010.519169

**Published:** 2010-10-08

**Authors:** Anton H Hosman, Henny C van der Mei, Sjoerd K Bulstra, Henk J Busscher, Daniëlle Neut

**Affiliations:** ^1^Department of Biomedical Engineering; ^2^Department of Orthopaedic Surgery, University Medical Center Groningen, Groningen, the Netherlands

## Abstract

**Background and purpose:**

Joint replacement with metal-on-metal (MOM) bearings have gained popularity in the last decades in young and active patients. However, the possible effects of MOM wear debris and its corrosion products are still the subject of debate. Alongside the potential disadvantages such as toxicity, the influences of metal particles and metal ions on infection risk are unclear.

**Methods:**

We reviewed the available literature on the influence of degradation products of MOM bearings in total hip arthroplasties on infection risk.

**Results:**

Wear products were found to influence the risk of infection by hampering the immune system, by inhibiting or accelerating bacterial growth, and by a possible antibiotic resistance and heavy metal co-selection mechanism.

**Interpretation:**

Whether or not the combined effects of MOM wear products make MOM bearings less or more prone to infection requires investigation in the near future.

Many young patients with painful coxarthrosis want to return to a high level of activity and require an implant that provides durability. The low wear rates of metal-on-metal (MOM) bearings have led to a resurgence in the use of MOM bearings (Wagner and Wagner 2000, Silva et al. 2005, Pollard et al. 2006, Vendittoli et al. 2007, Delaunay et al. 2008). 35% of all prostheses in the United States in 2006 (Bozic et al. 2009) and 16% of all prostheses implanted in Australia from 1999 through 2007 had MOM bearings (Graves et al. 2008).

Metal alloys used in MOM bearings degrade through wear, from corrosion, or by a combination of the two (Yan et al. 2006, Jacobs et al. 2008). Consequently, MOM bearings produce nanometer- to submicrometer-sized metal particles (Campbell et al. 1996, Doorn et al. 1998). The high number of these very small particles presents a large cumulative surface area for corrosion. The biological effects of these particles and their corrosion products in the human body are for the most part unclear. Since the renewed interest in MOM bearings, extensive research has been done to determine the consequences of local and systemic exposure to wear particles and accompanying biologically active corrosion products (Amstutz and Grigoris 1996). It is well known that metal debris can induce pathological changes such as the release of inflammatory cytokines from macrophages, histiocytosis, fibrosis, and necrosis (Basle et al. 1996, Granchi et al. 1998, Caicedo et al. 2008, 2009). Metal debris is also thought to be associated with hypersensitivity and osteolysis (Hallab et al. 2000, 2010, Goodman 2007b, Carr and DeSteiger 2008, Huber et al. 2009). However, there is very little literature on the bacteriological effects of these degradation products (Anwar et al. 2007, Hosman et al. 2009). It is therefore unclear whether they can influence the risk of infection.

The Australian and New Zealand joint registries have shown that between 9% and 15% of all total hip arthroplasty (THA) revisions are carried out because of infections related to the primary prosthesis (Rothwell et al. 2007, Graves et al. 2008). In cases of infection, bacteria adopt a biofilm mode of growth on the surface of the prosthesis, thus increasing the antibiotic resistance and resulting in major difficulties in treatment (Trampuz and Widmer 2006). Removal and replacement of an infected implant is usually required to eliminate the infection (Bozic and Ries 2005, Vincent et al. 2006). Recent research has suggested that particulate debris of any composition promotes bacterial growth by providing a scaffold for bacterial adhesion and biofilm growth (Anwar et al. 2007). On the other hand, high concentrations of metal ions have been shown to have bacteriostatic properties (Hosman et al. 2009).

Considering the paucity of publications on the effects of MOM particles on infection, we performed a review of the literature on the influence of MOM wear particles and their corrosion products on the risk of infection.

## MOM bearings

### History

First-generation MOM hip bearings include prostheses developed in the 1960s, such as the McKee-Farrar, the Ring, the Stanmore, and the Sivash prostheses (McKee and Watson-Farrar 1966, Ring 1968, Scales and Wilson 1969, Sivash 1969). Implants from this era survived for more than 25 years because of low wear rates and minimal osteolysis (Amstutz and Grigoris 1996). An analysis of 253 Ring MOM hip arthroplasties revealed a cumulative survival rate of 60% after 21 years (Bryant et al. 1991). The McKee-Farrar prosthesis performed equally well compared to the Ring arthroplasty, up to 26 years after initial implantation (Schmalzried et al. 1996). However, alongside these encouraging durability results, first-generation MOM studies also demonstrated metal wear debris in tissues adjacent to the implants, particularly in prostheses with loose components or impingement (Howie 1990). Furthermore, early MOM designs turned out to cause frequent early cup loosening (Schmalzried et al. 1996).

First-generation MOM articulations were commonly used until the mid-1970s. Most were abandoned in favor of metal-on-polyethylene (MOP) articulation. The main reason for this change was the introduction of the Charnley low-friction arthroplasty (Charnley 1972), which is still one of the most extensively documented hip prostheses in the literature (Callaghan et al. 2000, 2004, Wroblewski et al. 2007). Long-term results of first-generation MOM implants had boosted their popularity and led to the development of second-generation MOM implants in the early 1980s. In addition, polyethylene wear from MOP implants was then hypothesized to cause osteolysis around the implant (Wroblewski 1994, Oparaugo et al. 2001), which stimulated renewed interest in alternative bearings lacking a MOP interface, such as the second-generation MOM bearings (Brown et al. 2002).

Second-generation MOM implants have an improved bearing interface and are composed of alloys with an increased metal hardness. Newly produced bearings therefore have substantially lower rates of wear than highly cross-linked polyethylene (Fisher et al. 2006). On the whole, volumetric wear is reduced by 20- to 100-fold compared to MOP implants (Silva et al. 2005), suggesting that second-generation MOM prostheses may considerably reduce osteolysis (Sieber et al. 1999). Although medium- and long-term clinical results with MOM bearings appeared to have demonstrated excellent durability, recent studies have shown that MOM bearing systems are not refractory to osteolysis (Korovessis et al. 2006).

### Alloys

For implant alloys worldwide, two nomenclatures are used in parallel to each other (Table 1). First of all, ASTM standards with a capital “F” (medical devices) are practiced mainly in the USA (Holzwarth et al. 2005). Secondly, ISO standards are accepted in the rest of the world. The 2 approved cobalt-chromium (Co-Cr) alloys contain almost similar amounts of alloying elements. However, there is no information available on the exact content of certain elements such as nickel (Ni) and iron (Fe), as these standards only report maximum amounts.

**Table 1.** Chemical composition and mechanical properties of CoCr28M06 alloy required by standards ASTM F75 and ISO 5832-4

	ASTM F75	ISO 5832-4
Rm **^a^** (MPa)	655	665
Rp 0.2% **^b^** (MPa)	450	450
Cobalt, Co	balance to 100%	balance to 100%
Chromium, Cr	27–30%	27–30%
Molybdenum, Mo	5.0–7.0%	4.5–7.0%
Nickel, Ni	< 0.5%	< 1%
Iron, Fe	< 0.75%	< 1%
Silicon, Si	< 1%	< 1%
Manganese, Mn	< 1%	< 1%
Carbon, C	< 0.35%	< 0.35%

**^a^** Tensile strength is the stress at which a material breaks or permanently deforms.

**^b^** Yield strength is the stress at which a material begins to deform plastically.

## Wear products

### Wear

Wear in bearings can result in scratching and pounding of the surfaces, and eventually erosion of the material. Wear and corrosion are probably the major causes of release of metal into the tissues of MOM patients, and this poses a major concern regarding the use of MOM articulating devices. Linear wear rates range from 5 to 25 μm/year and are dependent on a multitude of factors such as the type of implant and positioning (Onda et al. 2008, Shimmin et al. 2008, Williams et al. 2008b) (Table 2).

**Table 2.** Wear rates of MOM bearing couples defined in different units

Type of wear	Wear rate	Method	Ref.
Linear wear rate of femoral heads per year	7.6 μm (range 2.9–13) to	Explanted implant(s)	Reinisch et al. (2003)
	250 μm (range 50–810)	Radiographic analysis	Stilling et al. (2009)
First year	25 μm	Explanted implant(s)	Sieber et al. (1999)
> 3 years	5 μm	Explanted implant(s)	Sieber et al. (1999
Volumetric wear rate of femoral heads per year	2.0 mm^3^ (range 0.55–3.7)	Explanted implant(s)	Reinisch et al. (2003)
	5.0 mm^3^ (range 0.22–22)	Explanted implant(s)	Willert et al. (1996)
Mass wear rate per year	17 mg (range 4.6–31)	Explanted implant(s)	Reinisch et al. (2003)
No. of particles per unit volume of wear per mm^3^	2.7 × 10^12^ – 1.5 × 10^13^	Pin-on-plate	Tipper et al. (1999)
Number of particles per 10^6^ cycles	4 × 10^12^ – 6 × 10^13^	Pin-on-plate	Tipper et al. (1999)
Number of particles per year	6.7 × 10^12^ – 2.5 × 10^14^	Explanted implant(s)	Doorn et al. (1998)

Note: Retrieval study data were obtained from patients undergoing revision of THAs with MOM bearing couples. Radiographic wear analysis was performed by analysis of digitized anteroposterior (AP) radiographs using a computerized method.

### Metal particles

Currently, tribological research is being conducted on the exact process of particle generation. Recent tribological investigations have revealed that a nano-crystalline layer 250–400 nm thick is formed on the MOM implant surfaces, containing (amongst others) proteins from the interfacial medium (Pourzal et al. 2009). Cracking of this nano-crystalline layer due to surface fatigue has been suggested to be the main mechanism of generation of wear particles (Buscher et al. 2005). Abrasive particles of MOM prostheses can cause local damage, resulting in an accelerated release of metal particles and ions (Yan et al. 2009).

Germain et al. (2003) emphasized that the nature, size, and amount of particles are important determinants of the biological effects of wear debris on cells in vitro. The reaction of the body is dependent on the characteristics of the particles (Table 3). Size analysis of particles isolated from failed arthroplasties has revealed a mean size of 660 nm for polyethylene particles in patients with MOP bearings (Minoda et al. 2008) and a size range from 51 to 116 nm for MOM debris (Doorn et al. 1996).

**Table 3.** Size and morphology of wear particles generated by a hip simulator or derived from tissue samples

Size and morphology	Particle generation and type of prosthesis	Method **^a^**	Ref.
80 ± 40 nm, round	Hip simulator with bearing ASTM F 799 and F1357 Co-28Cr-6Mo	TEM	Catelas et al. (2001)
50–90 nm, oval or needle-shaped	Hip simulator with bearing ASTM F 799 and F1357 Co-28Cr-6Mo	SEM	Tipper et al. (1999)
25–36 nm, round	Hip simulator with bearing ASTM F 799 and F1357 Co-28Cr-6Mo	TEM	Firkins et al. (2001)
< 50 nm (range 6–834 nm), oval or round	Periprosthetic tissue samples of 2 McKee-Farrar and one McMinn prosthesis	TEM	Doorn et al. (1998)
< 50 nm, irregular	Periprosthetic tissue samples of 640 Sikomet SM21	SEM	Brown et al. (2007)
40–120 nm, needle-shaped < 90 nm, round	Periprosthetic tissue samples of one Bicon plus Ti shell with polyethylene liner, Sikomet SM21 head, and SL-Plus stem	TEM, SEM and XPS	Milosev and Remskar (2008)

**^a^** TEM: transmission electron microscopy; SEM: scanning electron microscopy; XPS: X-ray photoelectron spectroscopy.

### Metal ions

The first report of visible corrosion of an orthopedic Vitallium implant (consisting of 60% Co and 20% Cr) was published by Weightman et al. in 1969. Before this clinical finding, it was generally accepted that Vitallium alloys provided adequate corrosion resistance (Scales et al. 1961). However, generation of metal ions is also evident in modern, more corrosion-resistant MOM alloys.

Various mechanisms of corrosion can cause the release of metal ions. One is fretting corrosion due to the movement of articulating surfaces causing damage to one or both surfaces. Disruption of the passive oxide layer causes direct contact with the metal surface, promoting fretting corrosion (Figure), which can be enhanced further by the presence of adhering microorganisms (Muthukumar et al. 2003).

**Figure:**
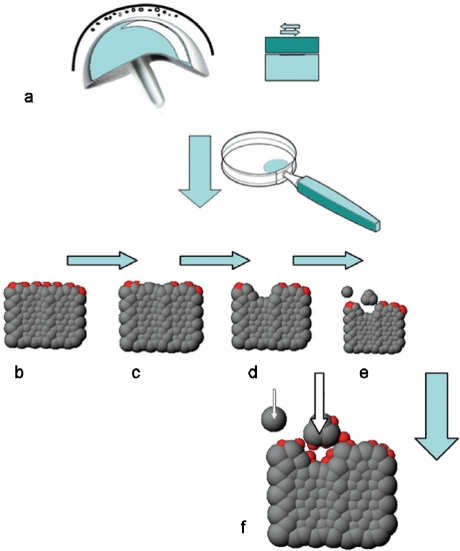
Schematic drawing illustrating: a. generation of wear particles; b. a metal alloy (gray scaffold) with an oxidized surface film on the upper surface (molecules marked in red); c. damage to the passive surface film (e.g. by scratching or pounding); d. occurrence of corrosion due to the lack of a protective layer; e. liberation of soluble compounds and wear particles; and f. repassivation of the surfaces including wear particles (arrows).

Metal ions of different valencies are released from Co-Cr alloys and their effects vary with the type of oxide compound they form (Virtanen 2006) (Table 4). Co^2+^ and Cr^3+^ ions predominate under physiological conditions because these ions are the most stable at neutral pH. However, no stable Co oxide exists and thus formation of soluble Co ions instead of solid Co oxides is favored. On the other hand, Cr^3+^ oxides are stable under physiological conditions. In vitro models have shown that the toxic effects of Co-Cr are probably due to Co^2+^ ions (Rae 1975, Garrett et al. 1983).

**Table 4.** Oxidation states of the elements in Co-Cr compounds

Co	Cr	Mn	Fe	Ni	Si	Mo
–1	–2	–3	–2	–1	–4 **^a^**	–2
+1	–1	–2	–1	+1	–3	–1
+2 **^a^**	+1	–1	+1	+2 **^a^**	–2	+1
+3 **^a^**	+2	+1	+2 **^a^**	+3	–1	+2
+4	+3 **^a^**	+2 **^a^**	+3 **^a^**	+4	+1	+3
+5	+4	+3	+4		+2	+4 **^a^**
	+5	+4 **^a^**	+5		+3	+5
	+6 **^a^**	+5	+6		+4 **^a^**	+6 **^a^**
		+6				
		+7 **^a^**				

**^a^** represents the most common oxidation states.

## Local concentrations

Metal ions may spread throughout the body. Ion levels have been measured in whole blood, serum, erythrocytes, and various solid tissues (Cobb and Schmalzreid 2006, Savarino et al. 2008, Lazennec et al. 2009). Serum Co levels are the most frequently reported metal ion concentrations, and they were found to be 5- to 6-fold higher in patients after MOM implantation than preoperatively (Lazennec et al. 2009).

Two well-received consensus papers have described the need to measure metal ion concentrations in the joint fluids of patients with MOM bearings (Amstutz et al. 1996, MacDonald et al. 2004) in addition to measuring metal concentrations in serum (Vendittoli et al. 2007, Savarino et al. 2008). Most research into metal ion concentrations does not, however, report the regional or local dissemination, as synovial biopsies are undesirable in otherwise healthy patients (Savarino et al. 2008).

There is therefore very little reliable information about the exact local concentrations of Co-Cr around prostheses (Table 5). Concentrations of Co ions have been found in the 6–6,000,000 μg/L range. This difference is not only related to alignment and the type of the prosthesis (Onda et al. 2008, Shimmin et al. 2008, Williams et al. 2008b) but also to improving detection methods (Dorr et al. 1990).

**Table 5.** Maximum levels of Co and Cr ions in local tissues of patients with a MOM implant

Sample	Prosthesis	Cobalt (μg/L)	Chromium (μg/L)	Method **^a^**	Ref.
Capsule	Cemented and loose	26,000	88,000	NAA	Evans et al. (1974)
Synovial fluid	Cemented and loose	250			
Capsule		22,000		NAA	Jones et al. (1975)
Femoral neck	Cemented	50,000	170,000	SES	Smethurst and Waterhouse (1977)
Acetabulum		170,000	170,000		
Lining from femoral stem		130,000	1,300,000		
Adjacent to articular surfaces		70,000	70,000		
Acetabular pelvic lining		200,000	200,000		
Capsule	Loose	6,000,000	1,500,000	AAS/NAA	Postel and Langlais (1977)
Synovial fluid	Cemented and loose	13,000	63,000	GSGSD	Dobbs and Minski (1980)
Capsule		63,000	327,000		
Granuloma		193,000	323,000		
Tissue (mid-femur)		6,900	5,500		
Synovial fluid	Cemented and loose	155	358	AAS	Davies et al. (2005a)
Synovial fuid	Cemented	199	347	GFAAS	Dorr et al. (1990)
Capsule	(well-fixed and loose)	3,971	1,465		
Fibrous membrane		2,451	1,634		
Synovial fluid	Cementless	1,015	617		
Capsule	(well-fixed and loose)	1,272	6,219		
Fibrous membrane		3,812	20,609		
Synovial fluid	Cemented and well-fixed	6	16	GFAAS	Brien et al. (1992)
Synovial fluid	Cemented and loose	152	238		

**^a^** NAA: neutron activation analysis; SES: spark emission spectroscopy; AAS: atomic absorption spectroscopy; GFAAS: graphite furnace atomic absorption spectrophotometry; GSGSD: gamma-spectroscopy with Ge-semiconductor detector.

Wear rates reported in recent hip simulator studies have turned out to show a close correlation with Co ion levels (Williams et al. 2008a), but measurement of ion levels in the lubricant of hip simulators resembles only certain aspects of the clinical situation. There is no free exchange between blood and synovial fluid (Dorr et al. 1990) and therefore wear products can accumulate in simulator systems, resulting in higher Co-Cr levels than would occur clinically (Table 6).

**Table 6.** Maximum levels of Co and Cr ions as measured with inductively coupled plasma mass spectroscopy in hip simulator lubricant, catogorized by head size of MOM implant and number of cycles

Head size	Co (μg/L)	Cr (μg/L)	Cycles (×10^6^)	Ref
55 mm	∼ 18,000	∼ 6,000	0.13	Leslie et al. (2008)
39 mm	∼ 12,000	∼ 4,000		
55 mm	10,915	3,675	0.13	Leslie et al. (2009)
39 mm	9,066	3,302		
36 mm	∼ 6,800,000	∼ 2,800,000	4	Williams et al. (2008a)
28 mm	∼ 12,000,000	∼ 8,000,000		

## Influence of MOM wear particles and corrosion products on the immune system

Orthopedic metals and their corrosion products modulate the activities of the immune system by influencing immunocompetent organs and cells by a variety of immunostimulatory and immunosuppressive mechanisms. Metal particles and ions spread throughout the whole body via lymph and blood, and have, for instance, been identified within macrophages in the liver and spleen (Urban et al. 2000, 2004). The long-term effects of the distant spread and accumulation of wear particles in the liver and spleen are unknown, but indicate that the immune system may be hampered. In this context, it should be emphasized that the mere presence of a foreign body itself already reduces the minimum number of bacteria required to cause infection (Zimmerli et al. 1982).

Another important issue is the hypothetical carcinogenesis due to MOM implants and its accompanying occupation of the immune system in combination with the use of immunosuppressive drugs. Ion release has been suspected to increase the risk of DNA damage (Savarino et al. 2000, Ladon et al. 2004) and it was recently found that Co-Cr nanoparticles can cause DNA damage across a cellular barrier (Bhabra et al. 2009). In addition, a reduction in the number of circulating cytotoxic CD8+ T-cells, which are responsible for destroying tumor cells, has been found in patients with a MOM implant (Mabilleau et al. 2008, Ogunwale et al. 2009). However, epidemiological studies do not allow conclusions regarding the incidence of cancer in patients with MOM implants (Nyren et al. 1995, Visuri et al. 1996, Dumbleton and Manley 2005) and they will not become available in the near future, as such studies would require thousands of patients to be followed for several decades (MacDonald et al. 2004).

### The spleen

The spleen is an important meeting point between antigenic information transported by the blood and the immunocompetent cells. Because of its central position in the bloodstream and its large blood supply of about 5% of the total blood volume per minute, the spleen will inevitably be exposed to corrosion products of MOM bearings. High concentrations of metal ions (375,000 μg/L Co and 200,000 μg/L Cr) have been shown to cause alterations in spleen architecture and depletion of T4 and B-cells. The immune system and its defense against bacteria may therefore become hampered by metal ions (Ferreira et al. 2003).

### The liver

The liver is part of the human immune system and it not only contains many immunologically active cells but also detoxifies environmental toxins. Metals cannot be eliminated from tissues by metabolic degradation, but only by renal or gastrointestinal excretion (Cobb and Schmalzreid 2006). There is evidence from a recent animal study to suggest that Cr ions can accumulate in the liver (Jakobsen et al. 2007). High levels of metal in the body may cause hepatocellular necrosis, as observed after acute ingestion of Cr^4+^ in humans. Clinically relevant concentrations of Cr^4+^ (10–25 μM) have been found to inhibit macromolecular syntheses in the liver (Keegan et al. 2007).

### Immunocompetent cells

Phagocytosing cells such as neutrophils are vital in the host defense against infection. These “first responders in microbial infection” are usually found in infected periprosthetic tissues. However, corrosion products of Co-Cr implant materials have been reported to inhibit the rapid release of reactive oxygen species required for bacterial killing by neutrophils (Shanbhag et al. 1992). In vitro studies have also shown that Co-Cr particles induce toxic effects after they are phagocytosed because of the drop in pH within the phagosome (Huk et al. 2004). Due to wear debris-induced granulocyte defects, patients with MOM implants may be predisposed to infection at the implant site (Zimmerli et al. 1982, 1984, 2004).

Degradation products, either in the form of metal ions or wear particles, can complex with local proteins and induce an allergic response comparable with a delayed-type hypersensitivity response (type IV), through activation of T-lymphocytes (Davies et al. 2005b, Goodman 2007a). The histological response in patients with MOM bearings is unique in its kind and is referred to as aseptic lymphocyte-dominated vasculitis-associated lesion (ALVAL) (Willert et al. 2005). In addition, a statistically significant reduction in circulating lymphocytes, in particular of CD8+ and T-cells, has been observed in patients with MOM bearings (Mabilleau et al. 2008, Ogunwale et al. 2009). However, at concentrations of Co and Cr below 5 μg/L, no such reduction was detected. No adverse clinical symptoms have been observed in patients with increased metal ion concentrations in serum (Hart et al. 2006).

## Influence of MOM degradation products on bacteria

### Heavy metal toxicity

Metal ions have been used for centuries to cure infections, and it is conceivable that wear products of MOM prostheses may be toxic to bacteria. There is in vitro and in vivo evidence that wear particles have toxic effects on human cells (Jones et al. 1975, Papageorgiou et al. 2007, Caicedo et al. 2008). In vitro research on influences of Co and Cr ions on bacteria have provided evidence of bacteriostatic effects (Anwar et al. 2007), hypothetically involving competition with Fe for uptake in the bacterial cell. Fe is an important nutrient element that is required by specific microbial species that use oxidation of elemental Fe or conversion of Fe^2+^ to Fe^3+^ as an energy source for their metabolism. Inhibition of Fe-dependent metabolic activities by Co ions has been shown to lead to growth retardation and cell death in Pseudomonas aeruginosa (Kothamasi and Kothamasi 2004).

Within the cells of tissues, nanoparticles are exposed to a series of oxidative mechanisms designed to destroy the foreign body, which leads to the generation of metal ions (Lundborg et al. 1992). Reactions with metal ions can lead to generation of free radicals: reactive oxygen species (ROS) and reactive nitrogen species (RNS), which can, in turn, cause cellular dysfunction. ROS and RNS are known to be involved in protein oxidation, leading to their degradation, lipid peroxidation, and DNA damage. Generation of ROS and RNS may, hypothetically, cause toxicity in bacteria as well.

## Bacterial growth and biofilm formation

Some recent studies have evaluated the influence of Co and Cr ions and Co-Cr particles on bacterial growth. Co and Cr concentrations of up to 20 μg/L and 9 μg/L, respectively, that have been reported to occur in serum showed no consistent influence on biofilm formation, but higher concentrations of 200,000 μg/L Co and 93,000 Cr μg/L statistically significantly reduced Staphylococcus aureus and CNS planktonic growth and biofilm formation (Hosman et al. 2009), suggesting that MOM bearings may be less prone to biofilm formation and subsequent infection. On the other hand, Anwar et al. (2007) showed that wear debris from MOM bearings accelerated the growth of planktonic bacteria. Aggregated particulate debris was suggested to promote growth by providing a scaffold on which biofilm can grow. In addition, it can be hypothesized that nanosized particles scattered throughout a biofilm would enhance the strength of its structure by working as a composite scaffold at the macroscopic level. Moreover, it is also possible that embedded particles in a biofilm might detach and act as carriers of biofilm throughout the joint and body.

## Heavy metal resistance

Bacteria have co-existed with abundantly found toxic heavy metals since the beginning of life. Thus, it was essential for bacteria to develop mechanisms of metal resistance. Bacterial resistance to metal toxicity is not only an environmentally important phenomenon but also has clinical implications for metal-bacterium interactions in MOM patients. Bacterial resistance mechanisms differ widely (Silver and Misra 1988) and are currently the subject of extensive studies. There are enzyme oxidases and reductases to convert metal ions from more toxic species to less toxic species (Caccavo Jr et al. 1994, Cervantes et al. 2001, Lloyd and Lovley 2001, Kamaludeen et al. 2003). There is also the possibility of binding heavy metals in the bacterial cell wall (Komeda et al. 1997). Blocking of cellular uptake is also an option by altering the uptake pathway. Once the toxic heavy metal has reached the cytoplasm, it can be pumped out again by a high-efflux system (Nies et al. 1989, Nies 1995). Efflux pumps are the major group of resistance systems currently known.

## Co-selection of antibiotic and metal resistance

There is growing concern that metal contamination may function as a selective agent in the proliferation of antibiotic resistance (Baker-Austin et al. 2006). It is hypothesized that antibiotic-resistant bacteria can be maintained in the environment owing to the co-regulation of resistance pathways (Baker-Austin et al. 2006, Wright et al. 2006). These co-selection mechanisms include co-resistance (with different determinants of resistance being present on the same genetic element) and cross-resistance (with the same genetic determinant being responsible for a conjoint resistance to antibiotics and metals). Co-resistance to multiple metals and antibiotics has been described in clinical isolates of Staphylococcus species (Ug and Ceylan 2003), but the most common co-resistance involves Cr, Pb, and penicillin. Co and Cr increase the sensitivity of staphylococci to penicillin, whereas sensitivity to tetracycline becomes less (Mnatsakanov 1967). The mechanisms behind this co-selection are currently being investigated.

It has been found that reduction of the permeability of bacteria causes Co and Ag resistance through a mechanism similar to that responsible for inhibiting β-lactam, ciprofloxacin, tetracycline, and chloramphenicol from entering the bacterium (Silver and Phung 1996, Ruiz et al. 2003). On top of this reduced permeability, a rapid efflux mechanism is also used to prevent Co, Cu, Zn, Cd, and Ni from entering the micro-organism, similar to the mechanism of resistance to ß-lactam, tetracycline, and chloramphenicol (Levy 2002, Nies 2003). The clinical incidence of co-selection mechanisms of resistance factors in pathogenic bacteria for antibiotics and heavy metals, and also their clinical implications, still remain unknown.

## Conclusions

Unfortunately, long-term clinical data on infection rates for MOM bearings are not yet available and therefore actual clinical influences on infection cannot be evaluated. To assess the clinical influence of bearing type on infection risk, studies will require thousands of patients to be followed for several decades. Such data may soon become available from national joint registries, and their evaluation will shed light on the net influence of bearing type on infection risk. However, this review suggests that wear particles and their corrosion products may have an influence on the risk of infection by hampering the immune system, by inhibiting or accelerating bacterial growth, and by possible antibiotic resistance and metal resistance mechanisms involving co-selection. Whether this influence results in an increase in clinical infection rates or in a decrease has not yet been investigated.
